# Mortality in dialysis patients may not be associated with ESA dose: a 2-year prospective observational study

**DOI:** 10.1186/1471-2369-13-40

**Published:** 2012-06-15

**Authors:** Lawrence P McMahon, Michael X Cai, Sanjeev Baweja, Stephen G Holt, Annette B Kent, Vlado Perkovic, Murray J Leikis, Gavin J Becker

**Affiliations:** 1 Monash University, Melbourne, Australia; 2 Department of Renal Medicine, Eastern Health, Melbourne, VIC, Australia; 3 Department of Renal Medicine, Royal Melbourne Hospital, Melbourne, VIC, Australia

**Keywords:** Haemoglobin, ESA, Dialysis, Prevalent, Mortality

## Abstract

**Background:**

Anaemia of chronic kidney disease increases the risk of death and adverse events, but can be managed using erythropoiesis stimulating agents (ESAs). However, recent evidence suggests that targeting a higher haemoglobin concentration ([Hb]) increases mortality risk, and both higher [Hb] targets and ESA doses have been implicated. Nonetheless, a causative role has not been demonstrated, and this potential relationship requires further appraisal in such a complex patient group.

**Methods:**

The relationship between the haematopoietic response to ESAs and patient survival in 302 stable, prevalent dialysis patients was explored in a prospective, single-centre study. Clinical and laboratory parameters influencing mortality and ESA resistance were analysed. Patients were stratified into 5 groups, according to their [Hb] and ESA dosage, and were followed for 2 years.

**Results:**

Little difference in co-morbidities between groups was identified. 73 patients died and 36 were transplanted. Initial analysis suggested a direct relationship between mortality and ESA dosage. However, Cox proportional hazards multivariate analysis demonstrated mortality risk was associated only with age (adjusted HR per year: 1.061, 95% CI 1.031-1.092), dialysis duration (adjusted HR: 1.010, 95% CI 1.004-1.016), peripheral vascular disease (adjusted HR: 1.967, 95% CI 1.083-3.576) and CRP (adjusted HR: 1.024, 95% CI 1.011-1.039). Mortality was increased in patients poorly responsive to ESAs (55.5%).

**Conclusion:**

ESA dose does not appear to contribute substantially to mortality risk in dialysis patients. Instead, age and co-morbidities appear to be the critical determinants. A poor response to ESAs is a marker of overall poor health status.

## Background

Erythropoiesis stimulating agents (ESAs) have been used to raise and maintain haemoglobin concentrations ([Hb]) since a link between anaemia, impaired quality of life, and adverse outcomes in patients with chronic kidney disease (CKD) was first suggested [1-3]. However, more recent studies have suggested that higher Hb targets (≥120 g/L) may paradoxically increase the rate of adverse vascular events [4-6]. In one recent randomised controlled trial in non dialysis-dependent CKD patients with diabetes and moderate anaemia (TREAT), the use of darbepoetin-alfa failed to reduce the risk of the composite end point of death and non-fatal cardiovascular events. More worryingly, it was also associated with an increased risk of stroke [7].

The mechanism for such an increase in the adverse event rate remains unclear, with suspicion falling on both the targeted [Hb] and ESAs themselves as causative agents [8]. The upper end of the target [Hb] range has been reduced in many countries; but a number of studies have also suggested that perhaps the absolute [Hb] is less important than the ESA dose [9-11]. However, it is important to recognise that the capacity of each of these trials to determine the true relationship has been limited by inherent trial design [12]. This fact was highlighted by an additional recent study, which found that after adjusting for multiple factors (including ESA dose), mortality actually reduced with increasing [Hb] in a prospectively followed incident cohort of Spanish haemodialysis patients [13].

This study was therefore designed to compare different ESA responsiveness patterns in a single-center cohort of prevalent dialysis patients, and to establish which factors influenced mortality. Whilst recognizing the inherent limitations of observational studies, we were able to explore more fully the relationship between [Hb], ESA resistance, and patient survival, incorporating the interplay of various clinical co-morbidities and laboratory parameters which are often unavailable to researchers in large-scale multicentre studies.

## Methods

### Population and categorization

This was a single-centre, prospective observational cohort study, approved by the Royal Melbourne Hospital Research and Ethics Committee. All prevalent dialysis-dependent patients (n = 596) were screened for eligibility between March and June 2003. Patients with a stable [Hb] (±5.0 g/L), no hospitalizations and an unchanged ESA dose (each for at least the prior 3 months), who were able to give informed consent were included.

**Figure 1:**
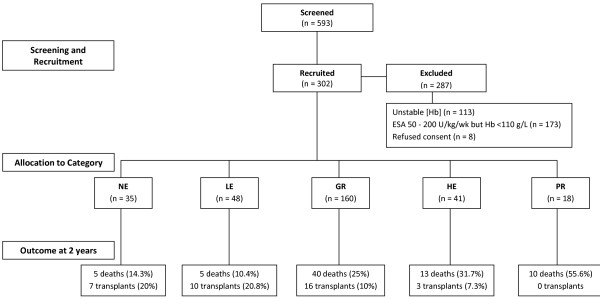
**Patient selection, categorization and outcome.** NE: No ESA; LE: Low ESA; GR: Good Responders; HE: High ESA; PR: Poor Responders.

ESA dose was expressed as IU/kg/week (darbepoetin alfa doses were converted as μg/kg/wk x 200). Patients were allocated to 1 of 5 groups (4 with [Hb] >110 g/L) according to ESA responsiveness as follows: NE (No ESA), patients with a [Hb] ≥110 g/L and not requiring ESA; LE (Low ESA), requiring minimal ESA (<50 IU/kg/week) with a [Hb] ≥110 g/L; GR (Good Responders), requiring 50–200 IU/kg/week to maintain a [Hb] ≥110 g/L; HE (High ESA), requiring >200 IU/kg/week to maintain a [Hb] ≥110 g/L; PR (Poor Responders), requiring >200 IU/kg/week, yet [Hb] <110 g/L (Figure 1).

### Clinical, demographic and laboratory assessments

Data collected at baseline included age, sex, aetiology of CKD, blood pressure, co-morbidities (coronary artery disease and/or congestive heart failure, peripheral vascular disease, prior cerebrovascular event, chronic airflow obstruction, treated hypertension and dyslipidaemia), body mass index (BMI), dialysis mode, and duration of end stage kidney disease (ESKD), including duration of dialysis and/or past renal transplantation.

Laboratory tests at baseline, 1 year and 2 years included [Hb], albumin, iron studies (ferritin and transferrin saturation, TSAT), and C-reactive protein (CRP). Serum vitamin B_12_, folate, and parathyroid hormone (PTH) were measured at baseline. Dose of dialysis for haemodialysis (HD) patients included both Kt/V and urea reduction ratio (URR); and for peritoneal dialysis (PD) patients a standard adequacy study was performed measuring total creatinine clearance and residual renal function. Changes in dialysis modality during the study were recorded.

### Statistical analysis

All analyses were performed by SPSS 18 (Chicago, Il, USA). This was an exploratory analysis examining the relationship between [Hb] and ESA dosage with particular regard to ESA dose (split into three groups, high, normal and low) and outcome, in patients with a similar [Hb]. The PR Group was added as control to exclude the effects of low [Hb] and high ESA. Data are displayed as mean ± standard deviation (SD), or median and IQR according to distribution and skew. Categorical variables were compared by the Chi-square test. Normally-distributed continuous variables were compared by ANOVA and for comparison of non-parametric continuous data Kruskal-Wallis test was applied. In a particular ESA category the difference between observations taken at baseline, 1 year or 2 years was assessed by *t*-test for normally distributed data, and for non-parametric data the Wilcoxon signed rank test was used. Kaplan-Meier survival curves were generated with patients censored if transplanted.

To compare the risk of mortality across groups the Cox multivariate proportional hazards model was used with adjustments for factors which could potentially alter the risk of mortality. The forward likelihood ratio (LR) method was used to estimate the influence of various covariates on ESA category-specific mortality risk. The covariates included ESA category followed by age, sex, cardiovascular disease, peripheral vascular disease (PVD), blood pressure, obesity, chronic airflow obstruction, cause of renal failure, dyslipidaemia, previous transplant, duration of ESKD, dialysis duration, dialysis modality, residual renal function, and the following baseline data: [Hb], ESA dosage, serum albumin, PTH, iron studies, CRP, and alkaline phosphatase concentrations. For model consistency, obesity was regarded as a categorical variable with a BMI cut-off at 30 g/m^2^, and the mode of dialysis was classified as HD or PD. The design of the study restricted having data on all these covariates at the time of death or follow-up. The primary outcome measure was death at 2 years. Secondary outcome measures included transplantation, change in [Hb] and change in ESA requirements.

## Results

Of 596 stable patients screened, 302 were enrolled and followed for 2 years (Figure 1). The most common cause of CKD was glomerulonephritis (40%) followed by diabetic nephropathy (18.5%). The highest percentage of patients with adult polycystic kidney disease was in the NE Group (17.1%) compared to 2.1%, 8.8%, 4.9%, and 5.6% for other groups respectively. Over 88% of patients were hypertensive, 34.8% had known coronary artery disease, 23.3% had peripheral vascular and 12.7% cerebrovascular disease. The median dialysis duration was 33 months. Only 6% had a BMI over 30. Overall, the severity of clinical co-morbidities differed little between groups. There were 244 haemodialysis (HD) and 58 peritoneal dialysis (PD) patients. Ten patients switched from PD to HD during the study (3 from NE, 4 from LE and 3 from GR Group). Most PD patients had significant residual renal function (mean creatinine clearance 16.9 L/wk), which was considered negligible in HD patients (urinary output <300 mL per day). There was no difference between groups in the duration of dialysis or ESKD, nor in the dose of dialysis received between groups (Table 1).

**Table 1:** Overall and group demographic and clinical data at study entry

	**Overall**	**NE**	**LE**	**GR**	**HE**	**PR**
**Age, ***years* *	60 ± 14	53 ± 14	57 ± 14	60 ± 15	64 ±12	69 ± 11
**Females, ***n (%)*	109 (36.1)	6 (17.1)	16 (33.3)	61 (38.1)	17 (41.5)	9 (50)
**Diabetes, ***n (%)*	56 (18.5)	7 (20.0)	15 (31.0)	23 (14.3)	8 (16.3)	3 (16.7)
**CAD, ***n (%)*	108 (35.8)	9 (25.7)	17 (35.4)	56 (35)	18 (43.9)	8 (44.4)
**HT, ***n (%)* *	267 (88.4)	26 (74.3)	44 (91.7)	146 (91.3)	34 (82.9)	17 (94.4)
**PVD, ***n (%)*	70 (23.3)	4 (11.4)	16 (33.3)	33 (20.6)	11 (26.8)	6 (33.3)
**CVA, ***n (%)* *	38 (12.7)	2 (5.7)	6 (12.5)	17 (10.6)	12 (29.3)	1 (5.6)
**COPD, ***n (%)*	29 (9.6)	4 (11.4)	3 (6.3)	13 (8.1)	7 (17.1)	2 (11.1)
**Dyslipidemia, ***n (%)*	118 (39.1)	8 (22.9)	24 (50)	60 (37.5)	17 (41.5)	9 (50)
**BMI ***kg/m*^*2*^ *	25.9 ± 5.6	27.1 ± 7.7	27.5 ± 5.9	25.1 ± 4.7	24.6 ± 4.4	28.9 ± 6.9
**Obesity, **n (%) *	18 (6)	3 (8.6)	6 (12.5)	6 (3.8)	0	3 (16.7)
**Previous transplant, ***n*(%)*	10 (28.6)	4 (8.3)	19 (11.9)	5 (12.2)	1 (5.6)	39 (12.9)
**HD, ***n (%)* *	244 (80.8)	24 (68.6)	30 (62.5)	135 (84.4)	38 (92.7)	17 (94.4)
**Duration of ESKD ***(months)*	34 (18–60)	49 (28–111)	26 (15–56)	34 (17–60)	31 (19–54)	31 (23–58)
**Time on dialysis ***(months)*	33 (18–58)	41 (27–88)	36 (18–66)	37 (22–59)	31 (23–58)	35 (18–61)

*BMI*: body mass index, *CAD*: coronary artery disease, *HT*: hypertension, *PVD*: peripheral vascular disease, *CVA*: past cerebrovascular accident, *COPD*: chronic obstructive pulmonary disease, Obesity: BMI > 30 kg/m^2^, HD: haemodialysis, ESKD: end stage kidney disease *: p < 0.05 for between-group analysis. Data are displayed as mean ± SD, n (%), or median (25^th^-75^th^ quartile), as indicated.

The NE Group was youngest, had the highest [Hb], and lowest prevalence of hypertension; whereas patients in the PR Group were older, more hypertensive, likely to be obese, and were less likely to have received a previous kidney transplant. The NE Group also had a higher baseline [Hb] and lower serum ferritin compared to other groups. In contrast, the PR Group had lower baseline [Hb] and serum albumin, and higher ferritin and CRP (Table 2).

**Table 2:** Laboratory parameters at the baseline (and end) of study as indicated

**Variable**	**Time**	**NE**	**LE**	**GR**	**HE**	**PR**
**Ferritin ***(μg/L)*	Initial *	202 (80–296)	429 (228–607)	347 (235–539)	469 (206–640)	516 (353–772)
	At 2 years *	181 (57–222)	285 (190–422)	389 (230–423)	288 (252–384)	239 (183–284)
**TSAT ***(%)*	Initial *	25 (20–38)	25 (19–37)	25 (20–33)	24 (19-27)	19 (16-23)
	*At 2 years*	26 (16–31)	26 (22–32)	22 (19-28)	22 (18-27)	17 (15-24)
**CRP ***(mg/dL)*	Initial *	4 (2-11)	6.5 (3.8-15.5)	5 (2-10)	8 (3-30)	17 (12-23)
	*At 2 years*	4 (2-9)	6.5 (3-14)	5 (2-10)	4 (2–13.5)	16 (5–37)
**Albumin ***(g/L)*	Initial *	38 ± 4	38 ± 5	37 ± 4	37 ± 4	34 ± 4
	*At 2 years*	37 ± 5	38 ± 5	40 ± 3	39 ± 4	37 ± 6
**PTH ***(pmol/L)*	*Initial*	23 (11–49)	23 (10–47)	22 (11–54)	28 (14–57)	44 (25–68)
**Vitamin B**_**12 **_*(pmol/L)*	*Initial*	317 (237–488)	396 (290–487)	373 (282–514)	362 (246–515.7)	274(217–454)
**Folate ***(nmol/L)*	*Initial*	42 (12–45)	19 (9–45)	35 (13–45)	45 (13–45)	39 (17–45)

Abbreviations as per text. Values given include median (and IQR), or mean ± SD. Initial values and those after 2 years are shown. *: p < 0.05 for between-group analysis at time point indicated.

**Figure 2:**
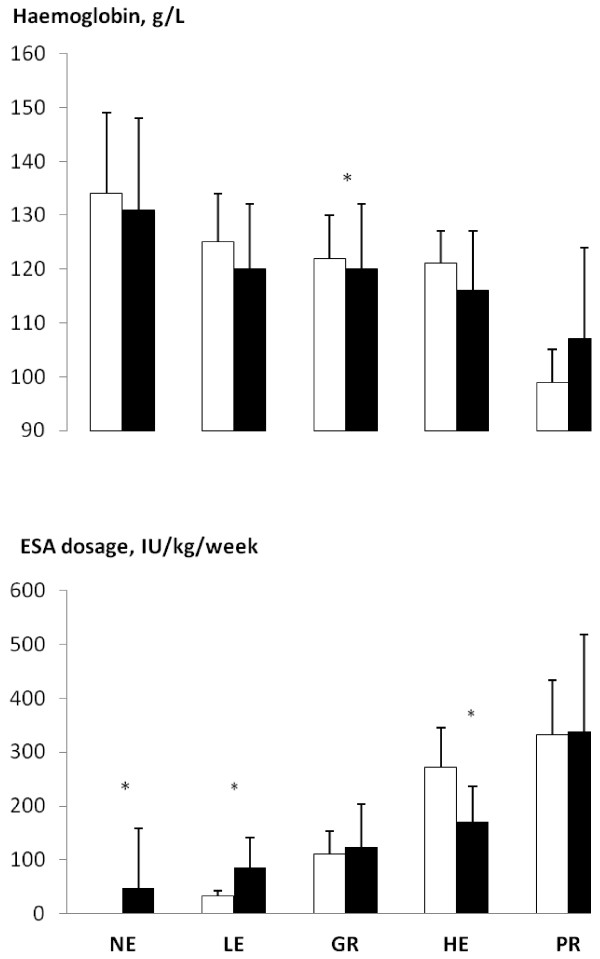
**Hemoglobin concentration and ESA dosage at baseline (white) and 2 years (black). Group coding as per Figure 1.** Significant differences were observed only in the GR Group. ESA dosage increased in the NE and LE Groups and decreased in the HE Group *: p < 0.05.

Of the 73 (24.2%) deaths in 2 years, the PR Group had the highest death rate (56%) followed by the HE, GR, NE and LE Groups respectively (Figure 1). At baseline and after 2 years, HD patients received a higher ESA dosage compared to PD patients despite no difference in age, BMI, haematinics (iron studies, vitamin B_12_ and folate), CRP or PTH levels. Death rates did not differ between HD and PD patients (23.9% vs. 22.4%, p = 0.48). The most common cause of death was a cardiovascular event (45.2%), followed by sepsis (17.8%). During the study period, 36 patients (11.9%) received a kidney transplant. The NE and LE Groups had the highest rates of transplantation (≥20%), while none of the PR patients were transplanted (Figure 1). There was no significant difference between baseline and 2-year [Hb] within or between groups, except in the GR Group (mean [Hb] 123 g/L versus 120, Figure 2). Survivors in the PR Group had a higher mean [Hb] at study end, although changes were not statistically significant (Figure 2). ESA requirements increased in both NE and LE Groups, and decreased in the HE Group; however the PR Group recorded the highest ESA dosage both initially and at 2 years (Figure 2, Table 2).

Over the 2-year period, the [Hb] remained relatively unchanged. The ESA dosage increased significantly in the NE, GR and HE Groups; however the PR Group remained on the highest doses (Figure 2). The poor hematopoietic response to ESAs persisted in the PR Group throughout the study and, in 2 years, only 3 of 18 patients had achieved a [Hb] >110 g/L.

**Figure 3:**
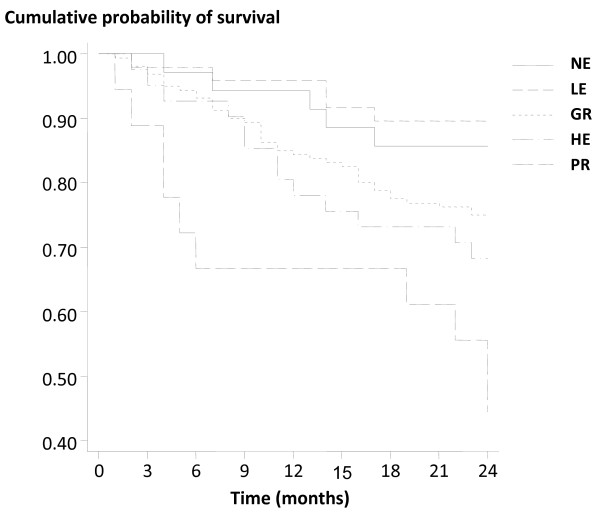
**Kaplan-Meier survival curve for all groups during the 2-year period.** Group coding as per Figure 1. P = 0.002.

The Kaplan-Meier survival analysis revealed a decreased cumulative survival in the PR Group compared to others, and suggested survival correlated with specified group (Figure 3). A Cox multivariate hazards analysis was undertaken, adjusted for all variables but specifically for age, CRP, duration on dialysis and PVD. Where death occurred the last known measure was carried forward to 2 years. The apparent increase in mortality risk in the PR group was found to be statistically associated with age, baseline CRP concentration, duration on dialysis and presence of PVD. In this model, [Hb], ESA dosage and, importantly, the assigned group category were not significant, suggesting they were not independent variables for mortality risk (Table 3, Figure 3). These data suggest that for every year increase in age, the 2-year mortality risk increased by approximately 6%, and by 2.5% for every 1 mg/dL increase in CRP. Additionally, each month on dialysis conferred a 1% increment in hazard ratio, and the presence of PVD doubled that risk (Table 3). Similarly, compared to the patients receiving low dose ESA (LE + GR Groups) those on high dose ESA (HE + PR Groups) had a decreased 2-year cumulative survival. This increased mortality risk was independently associated with age, baseline CRP, duration of dialysis and PVD, but not ESA dose. Even after excluding the PR Group from the model, the same variables were retained as significant within the model, but serum albumin became a significant contributor to the risk (Table 3).

**Table 3:** Cox proportional hazards model

**Variable**	**Adjusted mortality hazardsHR (95%CI)**	
	**All patients** (n = 203)	**Excluding PR Group** (n = 185)
Age (per year)	1.05 (1.02-1.08)*	1.04 (1.01-1.08)*
CRP (per unit rise)	1.02 (1.01-1.03)*	1.02 (1.01-1.03)*
Duration on dialysis (per month)	1.01 (1.00-1.01)*	1.01 (1.00-1.01)*
Peripheral vascular disease	1.75 (1.00-3.04)*	1.80 (0.97-3.25)
ESA dose	1.001 (0.996-1.006)	1.000 (0.997-1.004)
[Hb]	1.008 (0.962-1.056)	0.998 (0.947-1.052)
LE Group	0.37 (0.08-1.68)	0.46 (0.10-2.23)
GR Group	1.10 (0.38-3.17)	1.39 (0.41-4.75)
HE Group	0.96 (0.30-3.11)	1.07 (0.22-5.29)
PR Group	1.58 (0.49-5.09)	-
Test of model assumption	*X*_(4)_^2^ = 1.00, p = 0.910	*X*_(4)_^2^ = 0.33, p = 0.988

Adjusted hazard ratios of mortality for all patients (n = 203) and without the PR Group (n = 185).

A test of the proportional hazards assumption was undertaken and the models were deemed not to violate any assumption. *p < 0.05.

## Discussion

Findings from this study indicate that mortality in stable dialysis patients, when categorized by ESA dosage and [Hb], is related primarily to age, CRP, dialysis duration and peripheral vascular disease (PVD). The apparent association between mortality and higher ESA doses demonstrated in the Kaplan Meier regression curves (Figure 3), and which has also been observed in interventional studies [4-7,14] proved to be spurious as no direct relationship was found when other specific factors were taken into account (Table 3). Findings remained constant whether the PR Group (with the highest mortality risk, the highest ESA dosage and the lowest [Hb]) was included or not.

Age is known to be an independent and powerful, if unavoidable, predictor of death for patients with ESKD, and is also significantly associated with PVD especially amongst non-diabetic CKD patients [15-17]. Peripheral vascular disease is itself an independent determinant of all-cause mortality and cardiovascular events in ESKD. The presence of PVD in haemodialysis patients doubles the risk of hospitalization for major cardiovascular events, and increases the risk for all-cause hospitalizations by 19% [18,19]. Chronic inflammation is also associated with increased morbidity and mortality in patients receiving dialysis, and the inter-relationship between inflammation, inadequate nutrition and a poor response to ESA is well recognised [20-22]. Our study thus adds weight to the hypothesis that it is these well-identified risk factors of age, dialysis duration, vascular disease and inflammation which appear to determine mortality outcomes in a dialysis cohort. In contrast, hyporesponsiveness to ESAs appears to be simply a surrogate marker of poor underlying overall health.

The recently published ANSWER Study [13] comprised incident haemodialysis patients, whereas all patients in the current study were prevalent dialysis patients, many of whom had been on dialysis for over four years, and almost 20% of whom were on peritoneal dialysis. However, patients in each study exhibited substantial cardiovascular co-morbidity, and findings were similar, except that the ANSWER Study specified one ESA dosage (between 8000 and 16000 IU/week) as an independent predictor of mortality. However, higher ESA doses were not similarly identified, and it is difficult to reconcile this result with anything other than a statistical anomaly. Other factors potentially contributing to poor ESA responsiveness in our study, including dialysis dose, hematinic studies and parathyroid hormone levels, were specifically looked for and found not to be associated with mortality. Such variables also did not differ between groups. Our findings corroborate the findings of the ANSWER Study, in that neither the high ESA dosage nor [Hb] directly related to mortality in this patient group.

Is it possible to reconcile these findings with the larger randomized controlled trials (RCT) which have highlighted an apparent association between a reduced hematopoietic response to ESA and greater risk of an adverse outcome? Unfortunately, further analysis from these studies is limited by lack of data on other issues relating to individual patients’ health status, many of which can influence achieved [Hb] and ESA dose [4-7,14,23-26]. Nonetheless, relative differences between these interventional studies, including the nature of the patient cohorts, outcomes and endpoints, and the variable dose of ESAs required, highlighted features consistent with the current study. In particular, patients with greater cardiovascular co-morbidity generally had a higher mortality rate and used substantially more quantities of a given ESA to achieve the target [Hb]. As stated, none of these RCTs could demonstrate more than an associative relationship between ESA dose and mortality, and the observational nature of our study similarly precludes causation. However, the findings do suggest caution in establishing an overly simplistic explanation between death and the use of ESAs.

Thrombosis is a recognized and concerning complication of ESA treatment. It was first identified as a risk factor for access thrombosis in the Canadian placebo-controlled study [27], and subsequently confirmed in the Normal Hematocrit Study [6], amongst others. It was also recently confirmed as a risk factor for (mainly) non-haemorrhagic stroke in the TREAT Study [7]. Thrombosis itself was not studied as an endpoint in the current study and it might have been useful in determining the influence of ESA dose and/or [Hb] in vascular access thrombosis. Whether it would have been relevant in assessing thrombotic stroke risk is arguable. Although stroke was clearly evident as a risk factor in the TREAT Study, it took 4000 diabetic patients, mostly with known cardiovascular co-morbidities, for it to be identified as such. A recent placebo-controlled study of ESA use in acute ischemic stroke corroborated these findings [28]. It therefore remains uncertain whether ESA dose and/or Hb are associated with thrombotic stroke in a dose and/or concentration-dependent fashion, although ESA use itself must be accepted as inherently thrombogenic.

The [Hb] used to define target groups was higher than current targets [29], but was within standard parameters when the study was performed. We believe this is one of the study’s strengths, since any direct effect of ESAs or [Hb] should have been accentuated by such a strategy. In addition, the mortality rate for the group overall did not appear materially different to previously reported studies, suggesting that the target [Hb] issue was not of great relevance. Furthermore, the cause of death showed no statistical differences between groups, although this could well relate to the limited numbers.

Despite a higher mean [Hb] (134 g/L) in the NE Group, mortality was no higher, and possibly less, compared with other groups. At least one recent report suggests that a naturally-occurring [Hb], even at physiological concentrations, is not associated with a higher mortality risk [30]. These findings are also supported by our data. The two-year mortality was highest in the PR group. After adjusting for risk factors, it became evident that these patients were older, had been on dialysis longer, had worse PVD and a higher CRP. Such a high mortality index is difficult to resolve with the small numbers of patients in this group – only eight patients were left at study completion. However, even when the PR group was excluded from the analysis, the factors most clearly associated with mortality in groups with comparable [Hb] but different ESA requirements were unchanged.

There are acknowledged limitations of this study. First, it is observational and limited conclusions only can be drawn. Secondly, the sample size is relatively small, and varied substantially between groups. The associated lack of power might well have affected the capacity to discriminate between individual group results. Thirdly, patients were followed for only two years, with a relatively low mortality index in most groups. However, the magnitude of the mortality hazard risk associated with other co-morbidities appears to be much larger than any ESA specific effect, and since the results are consistent internally (between groups) and externally (compared to other studies), we feel the results are valid and contribute to our understanding of the effects of ESA use in the dialysis population. Finally, using only stable patients excluded many patients from the cohort. Although adding to patient numbers and possibly providing more comprehensive data, to have studied those with cyclical or rising ESA needs would have required a different approach to both baseline and follow-up.

## Conclusion

In conclusion, in this prospective two-year cohort study, age, underlying inflammation, duration of dialysis, and the presence of PVD were significantly associated with mortality in dialysis patients. No independent association was found with group, [Hb] or ESA dose, suggesting that co-morbidity and age are the key players determining longevity in this group of patients.

## Competing interests

LM has received unrestricted research grants from Roche and Amgen. Other authors have no competing interests.

## Authors’ contributions

LM, VP and GB conceived and planned the study. MC, MJ and AK collected and organized patient data. MC, SB, VP and SH contributed to statistical analysis. LM, MC, SB, SH and GB wrote and revised the manuscript. All authors read and approved the final manuscript.

## Pre-publication history

The pre-publication history for this paper can be accessed here:

http://www.biomedcentral.com/1471-2369/13/40/prepub
